# O003. Red ear syndrome: a new form of trigeminal autonomic cephalalgia?

**DOI:** 10.1186/1129-2377-16-S1-A127

**Published:** 2015-09-28

**Authors:** Carlo Lisotto, Federico Mainardi, Ferdinando Maggioni, Giorgio Zanchin

**Affiliations:** Headache Centre, Department of Neurosciences, University of Padua, Padua, Italy; Headache Centre, Hospital of Venice, Venice, Italy

## Background

Red ear syndrome (RES) is characterized by unilateral reddening of the external ear with associated pain and burning sensation. Some authors proposed a distinction between an idiopathic form, more commonly seen in young people and associated with migraine, and a secondary type, occurring more frequently in adults in association with cervical disorders[1]. This newly described condition lacks definition in terms of aetiology, pathophysiology and treatment. For this reason it has yet to be included in the International Classification of Headache Disorders (ICHD-3 beta). We report the case of a female patient with idiopathic RES, who responded to pregabalin 300 mg daily.

## Materials and methods

Following the diagnostic criteria proposed by Lambru et al[2], we diagnosed with primary RES in a 30-year-old female, whose symptoms started when she was 28.

## Results

The pain was unilateral, strictly left-sided, felt maximal on the ear lobe, radiating towards the ipsilateral mandible, cheek and eye, not infrequently in association with conjunctival injection and mild lacrimation; it was reported as severe. The attacks were consistently associated with marked ear reddening. The pain was described as a burning sensation and simultaneously as stabbing and jabbing. The duration of each RES episode could vary, ranging from 30 to 90 minutes. As well the attack frequency was widely variable, from 2-3 daily to one every 5 days, exclusively occurring during the daytime. The MRI of the brain (including angiographic study) and of the neck was within normal limits. As for treatment, our patient obtained a significant reduction of pain using pregabalin 300 mg daily.

## Conclusions

Some authors have suggested that RES could be considered a form of trigeminal autonomic cephalalgia (TAC) on the basis that both have a similar phenotype characterized by short-lasting attacks of unilateral pain, associated with cranial autonomic features[3]. The majority of RES described in the literature are primary but secondary RES has been reported. The underlying disorder can encompass mainly upper cervical spine lesions and temporo-mandibular joint dysfunction. Several different drugs have been tried in RES patients, but most of them seem to produce a marginal benefit. Gabapentin has been the most widely used medication in subjects with RES. This condition has been described in some patients in association with TACs, except for hemicrania continua, thereby supporting the possible nosological and pathophysiological link between RES and TACs. We propose RES to be included in the ICHD-3 beta Appendix in the TACs chapter.

Written informed consent to publication was obtained from the patient(s).
